# Population‐based analysis of perioperative chemotherapy use, interventions requiring hospitalization and atheroembolic events among patients with non‐metastatic muscle‐invasive bladder cancer

**DOI:** 10.1002/cam4.3805

**Published:** 2021-03-12

**Authors:** Tarik Benidir, Jaime Herrera‐Caceres, Christopher Wallis, Katherine Lajkosz, Neil Fleshner

**Affiliations:** ^1^ University of Toronto Toronto ON Canada; ^2^ Vanderbilt University Nashville TN USA

**Keywords:** bladder cancer, complication, intervention, perioperative chemotherapy, thromboembolism

## Abstract

**Introduction:**

Utilization of neoadjuvant chemotherapy (NC) in muscle invasive bladder cancer (MIBC) is increasingly recognized as standard of care but trends of use in Ontario remain unknown. Currently, there remains knowledge gaps regarding the effects of perioperative chemotherapy on the rates of interventions requiring hospitalization (IRH) and atheroembolic events (ATEs).

**Methods:**

We conducted a population‐based retrospective study within the province of Ontario over 16 years. Patients with non‐metastatic MIBC receiving surgery only or planned for perioperative chemotherapy were included. Primary outcomes included 2‐year IRH and ATE rates. Univariate/multivariate analysis was used to identify predictors associated with IRHs and ATEs. Cochrane‐Armitage was used to assess treatment trends over time.

**Results:**

Our study included 3281 patients. RC alone occurred in 2030 (60.9%), NC in 974 (29.6%) and adjuvant chemotherapy in 8.4% (*n* = 277). A total of 490/974 (50.3%) patients whom initiated NC with RC intent failed to undergo RC. This improved to 20.5% by 2015 (*p* < 0.001). Use of NC increased by an absolute value of 33% (*p* < 0.001). Overall, 4.2% of patients experienced IRHs and 11.5% ATEs. On multivariate analysis, advanced age and Charlson index score (CI) were strong predictors of outcomes, not timing of perioperative chemotherapy (*p* < 0.05.)

**Conclusion:**

A total of 29.6% of MIBC patients are planned for NC with 20.5% not progressing to their surgery. Use of NC has substantially increased over time. IRHs and ATEs remain stubbornly high at 4.2% and 11.5% respectively. Older age and higher CI scores are the strongest predictors of IRHs and ATEs (*p* < 0.05), not perioperative chemotherapy.

## Graphical abstract

In the Province of Ontario, trends suggest an increase in the adoption of neoadjuvant chemotherapy from 3% in 2002 to 35% in 2015 (*p* < 0.01). Radical cystectomy without chemotherapy still remains the largest utilized treatment approach at 54%. A substantial proportion of patients, particularly the elderly and comorbid, fail to progress to surgical intervention following initiation of neoadjuvant chemotherapy. Although this number has substantially improved (50.3% to 20.5%). Rates of ATEs and IRH’s remain stubbornly high, are not associated to timing of chemotherapy but are rather associated to unmodifiable patient risk factors; notably older age (*p* < 0.001) and in certain instances higher Charlson Index score.

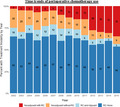

## 1 INTRODUCTION

Level one evidence supports neoadjuvant chemotherapy (NC) prior to radical cystectomy (RC) for muscle‐invasive bladder cancer (MIBC). The data in support of this recommendation are largely based on disparate‐regimen trials^1^, ^2^, ^3^, ^4^, ^5^ and include some results which did not reach statistical significance.^3^ Nonetheless, a meta‐analysis of these trials did show a 5‐year OS benefit of 5% and is the basis for guideline recommendations.^5^ Randomized studies exploring adjuvant chemotherapy (AC) are underpowered and contain methodological flaws and as a result, are not considered standard of care.^6^, ^7^, ^8^, ^9^, ^10^ Despite a paucity of data supporting AC in the MIBC space, many oncologists consider this approach reasonable for patients with pathologic high‐risk disease following RC. Despite current evidence, NC prior to RC is not widely adopted, although trends seem to suggest increased utilization.^11^, ^12^, ^13^

Chemotherapy is associated with a significantly increased risk of atheroembolic events in bladder cancer patients.^14^, ^15^, ^16^, ^17^, ^18^, ^19^ Numerous additional factors contribute to the risk of thromboembolic events in this patient population including the following: age, frailty, smoking history, pelvic surgery, immobility, obesity, and cardiovascular comorbidities.^11^, ^12^, ^13^, ^16^ As a result, approximately 8–22% of patients receiving standard of care therapy for MIBC develop a thromboembolic event.^20^, ^21^, ^22^ Current literature contains significant gaps in our knowledge about timing of chemotherapy and its induced adverse events in MIBC patients. Many of the data emanate from randomized trials which represent a selected group of patients.^1^, ^2^, ^3^, ^4^, ^5^ Secondly, many population‐based studies narrowly focus on venous thromboembolisms creating a deficit in knowledge regarding arterial‐based thromboembolism; a condition with often more profound risk of death, health‐related costs, and disability.^23^, ^24^ Thirdly, the risks of both arterial and venous thromboembolic events, leading to an intervention requiring hospitalization (IRHs), have yet to be explored. IRHs are particularly important as they add significantly to cost of care as well as long‐term morbidity and mortality.^12^, ^25^, ^26^

Our objective in this study was to assess the latest trends for the use of perioperative chemotherapy in Ontario, Canada, a jurisdiction with publicly funded fee‐for‐service health care. We also wanted to provide a depiction of real‐world risks for IRHs and arterial/venous thromboembolic events (ATE) among MIBC patients treated with NC versus AC.

## 2 MATERIAL AND METHODS

**FIGURE 1:**
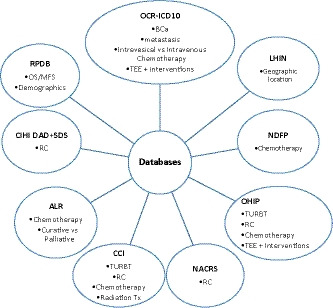
Databases used for patient accrual

We conducted a population‐based retrospective study within the province of Ontario utilizing hospital, billing, and procedural data via the Institute of Clinical Evaluative Sciences. This institute contains all major databases in the Ontario healthcare system and is extensively utilized for population‐based healthcare studies. The datasets utilized for this project are shown in Figure 1.

Cohort assembly included patients with bladder cancer and a history of a transurethral bladder tumor resection (TURBT). When multiple TURBT’s had occurred for a single patient, the TURBT preceding and closest to the starting date of intravenous chemotherapy or RC (whichever came first) was considered *the Index TURBT*. Patients required either a history of intravenous chemotherapy and/or a RC intervention and in no particular order. For the NC cohort, patients were required to initialize the first cycle of intravenous chemotherapy within four months or less of the *index TURBT* date. Of note, if no RC occurred after index TURBT, the patient was deemed neoadjuvant as long as intravenous chemotherapy was started within 4 months or less of the *index TURBT date*. For AC, patients would have required to initialize the first cycle of intravenous chemotherapy within 4 months or less of the RC date.

We utilized four criteria to exclude patients whom received palliative intravenous chemotherapy for advanced/metastatic disease. Any patient whose chemotherapeutic intervention occurred >4 months after the index TURBT date or RC date was deemed palliative and excluded. Any patient with an International Classification of Disease (ICD‐10) or Canadian Classification of Health Interventions (CCI) code for metastatic disease within 7 months of index TURBT date was excluded. Any patient whose coding was “palliative” within 12 months following index TURBT was excluded. Accuracy of “palliative” coding with the Cancer Activity Level Reporting (ALR) database has been validated.^27^ In addition, any patient receiving six cycles of intravenous chemotherapy within 7 months following *index TURBT* date was excluded as a maximum of four is considered standard of care in the adjuvant/neoadjuvant setting. Finally, we excluded patients who received chemotherapy for other cancers, intravesical chemotherapy, or trimodal radiotherapy.

Patient accrual date started March 1, 2002, and ended March 31, 2016 (to allow a 2 year follow‐up), for the primary outcomes in question. Outcomes were evaluated from March 1, 2002, until March 31, 2018. Primary outcomes included 2‐year IRH and ATE rates. A few examples of IRHs include (coronary catherization, bypass grafts, ventricular assisted devices, embolectomy/thrombectomy, ruptured aneurysms, etc.) A few of examples of ATEs include myocardial/cerebral infarction, non‐traumatic intracerebral hemorrhage, renal embolisms, and pulmonary embolisms. The complete list of included IRHs and ATEs considered for this study is large and therefore, provided in Appendix S1.

For patients receiving surgery alone, outcomes were assessed up to 2 years following the RC date. For patients receiving both RC and intravenous chemotherapy, the start date to evaluate outcomes was up to 2 years from whichever therapeutic modality came first following *index TURBT* (i.e., either the 1st cycle of chemotherapy following *index TURBT* or the RC date). For patients receiving intravenous chemotherapy only (i.e., RC was intended but did not occur), the start date to evaluate outcomes was at the onset of the first cycle of intravenous chemotherapy following *index TURBT date*. Baseline characteristics including age, gender, comorbidities, Charlson Index (CI) Score, and year of treatment were gathered to identify confounding factors.

We utilized a Cochrane–Armitage test of trend to identify significant treatment changes over time. Owing for this long period of observation, we further divided these years of observation into two eras to account for delays in treatment adoptions. Era 1 was (2002–2008) starting at the time of the *Grossman et al* paper, and Era 2 was (2008‐onwards). Univariate and Cox proportional multivariate analysis was used to identify statistically significant predictors of IRH’s and ATE outcomes over this 16‐year period and also when stratified by eras.

## 3 RESULTS

### 3.1 Baseline demographics

Table 1 displays the baseline demographics of the cohort. A total of 3281 patients were identified. *RC*‐*only* was delivered in 2030 (61.9%), *NC* was initiated in 974 (29.6%) and *AC* was delivered in 8.4% (*n* = 277). Over this 16‐year period of observation, a substantial proportion of patients 490/974 (50.3%) whom initiated NC with RC intent failed to actually undergo the surgical procedure (*NCnoRC*). Patients receiving RC‐only or those in the NCnoRC cohort were on average more elderly and comorbid than those completing multimodal treatment (*p* < 0.001; Table 1).

**TABLE 1:** Baseline patient demographics according to treatment modality

Demographics		RC‐only	NCnoRC	*p*	NC+RC	*p*	RC+AC	*p*
Number (n)		2030	490		484		277	
Age (%)				0.118		**<0.001**		**<0.001**
<64	24.1		28.6		43.4		44.0	
65‐74	35.1		32.7		37.2		37.5	
>75	40.8		38.8		19.4		18.4	
Male gender (%)	75.4		76.7	0.566	76.2	0.732	78.3	0.314
Comorbidities (%)								
COPD	26.7		28.6	0.435	20.5	**0.005**	23.8	0.344
CHF	7.6		10.4	**0.050**	3.7	**0.003**	4.0	**0.038**
DM	27.7		27.3	0.925	19.0	**<0.001**	16.2	**<0.001**
Asthma	11.2		11.8	0.740	8.3	0.073	8.7	0.246
MI	6.3		7.3	0.461	5.6	0.622	5.8	0.834
Crohns	0.9		1.0	0.988	1.0	0.969	1.1	1
HTN	65.2		64.9	0.951	52.5	**<0.001**	58.5	**0.035**
Charlson Comorbidity Index (CI)	0.73		0.74	**<0.01**	0.38	**<0.001**	0.61	**<0.001**

Patient demographics: all *P* values are compared to the RC‐only group. Statistically significant findings are emboldened. (1) RC‐only (patients undergoing Radical Cystectomy with no chemotherapy), (2) NCnoRC (initiating chemotherapy with RC intent but failing to progress to RC), (3) NC+RC (receiving neoadjuvant chemotherapy and subsequently Radical Cystectomy), (4) RC+AC (undergoing Radical Cystectomy followed by adjuvant chemotherapy).

### 3.2 Perioperative chemotherapy time trends

**FIGURE 2:**
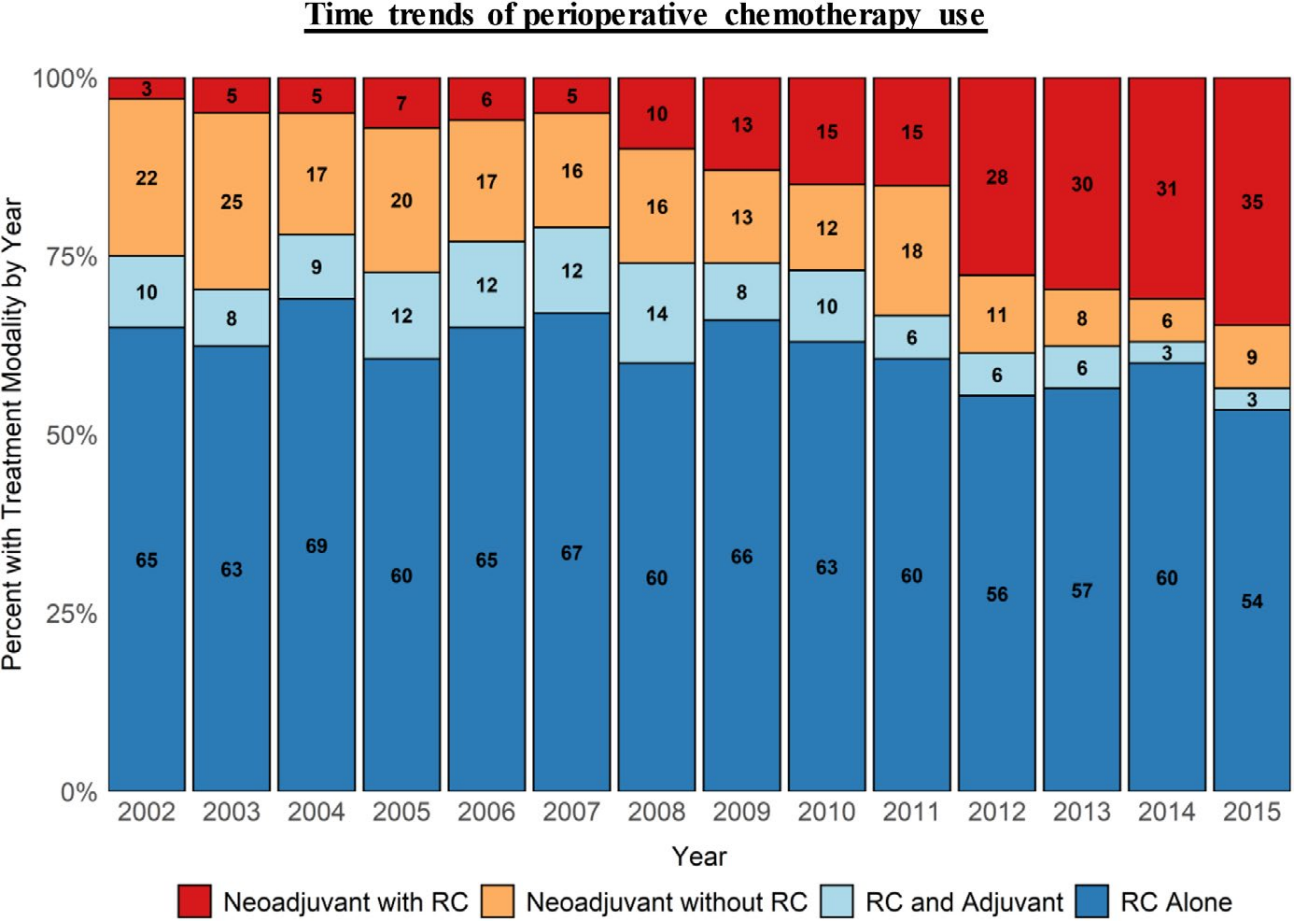
Time trends: there is a statistically significant change in all treatment modalities over time (*p* < 0.001). NC+RC increased from 3% in 2002 to 35% in 2015 (*p* < 0.001) with AC decreasing to 3% from 10%.

Using the Cochrane–Armitage test for trend, all treatment arms demonstrated statistically significant changes in treatment modalities over time (*p* < 0.001). Utilization of NC+RC rose from 3% in 2002 to 35% in 2015 (*p* < 0.001). The proportion of NCnoRC patients significantly decreased with time but still remained noteworthy (20.5% in 2015). Regardless of year, RC‐only remained the most chosen treatment modality, although its frequency had statistically declined (65% to 54%) (*p* < 0.001). Finally, utilization of AC had also declined from 10% to 3% (*p* < 0.001) (Figure 2).

### 3.3 IRHs and ATEs according to the use and/or timing of perioperative chemotherapy

Among the entire cohort of patients over a 2‐year follow‐up, 137 developed IRHs (4.2%) and 376 experienced ATEs (11.5%). Utilizing univariate analyses to stratify according to treatment modality, the overall IRH rates ranged from 2.5% to 4.6% (*p* = 0.17), and ATE rates ranged from 8.2% to 12.5%. The only subgroup on univariate analysis that displayed a higher rate of ATEs was the RC‐only group versus the NCnoRC group (12.5% vs 8.2%, *p* = 0.024; Table 2).

**TABLE 2:** Crude rates of IRH and ATEs

Treatment modality	ATE (n)	ATE (%)	IRH (n)	IRH (%)
RC‐only	253	12.5^a^	94	4.6
NCnoRC	40	8.2^a^	15	3.1
NC+RC	56	11.6	21	4.3
RC+AC	27	9.7	7	2.5
Total	376	11.5	137	4.2

Crude rates of IRH and ATEs across all four substrata.

^a^ The RC‐only group had significantly higher rates of ATE compared to the NCnoRC group (*P* = 0.024).

Upon multivariate analyses, the association of higher ATEs in the RC‐only vs the NCnoRC group is preserved. However, additional predictors of ATEs emerge including advanced age and CI. With respect to IRHs, advanced age and treatment era significantly predict risk (Table 3).

**TABLE 3:** Multivariate analysis of IRHs and ATEs comparing RC‐only to all other modalities involving chemotherapy

Characteristics	ATE		IRH	
	HR (95% CI)	*p* value	HR (95% CI)	*p* value
RC‐only vs NCnoRC	1.58 (1.12‐2.22)	**0.008**	1.47 (0.84‐2.56)	0.17
Male Gender	0.96 (0.73‐1.25)	0.775	1.33 (0.83‐2.15)	0.24
Age 65‐74	**2.03 (1.43‐2.90)**	**<0.0001**	**1.89 (1.08‐3.30)**	**0.026**
Age >75	**2.24 (1.58‐3.17)**	**<0.0001**	**1.92 (1.11‐3.34)**	**0.020**
CI	**1.10 (1.01‐1.20)**	**0.04**	1.04 (0.89‐1.21)	0.59
Era 2 (2008‐)	1.02 (0.81‐1.29)	0.85	1.44 (0.97‐2.13)	0.07
RC‐only vs NC+RC	1.02(0.74‐1.36)	0.91	1.16 (0.71‐1.92)	0.86
Male Gender	1.00 (0.77‐1.30)	0.98	1.15 (0.74‐1.80)	0.53
Age 65‐74	**1.74 (1.27‐2.39)**	**0.0006**	1.56 (0.94–2.59)	0.08
Age >75	**1.98 (1.44‐2.71)**	**<0.0001**	**1.70 (1.02‐2.80)**	**0.04**
CI	1.08 (0.99‐1.19)	0.08	1.06 (0.92‐1.24)	0.38
Era 2 (2008‐)	0.94 (0.74‐1.20)	0.60	**1.65 (1.08‐2.51)**	**0.018**
RC‐only vs RC+AC	1.14 (0.76‐1.69)	0.50	1.64 (0.75‐3.57)	0.21
Male Gender	0.98 (0.75‐1.29)	0.89	1.23 (0.76‐2.00)	0.39
Age 65‐74	**2.17 (1.51‐3.15)**	**<0.0001**	**1.86 (1.02‐3.34)**	**0.04**
Age >75	**2.65 (1.83‐3.77)**	**<0.0001**	**2.03 (1.14‐3.61)**	**0.015**
CI	**1.11 (1.01‐1.21)**	**0.025**	1.07 (0.92‐1.25)	0.32
Era 2 (2008‐)	0.89 (0.70‐1.12)	0.32	**1.58 (1.04‐2.39)**	**0.030**

Multivariate analysis for RC‐only versus all chemotherapy cohorts. Advancing age and CI was a significant predictor of ATEs. Advancing age and treatment era was a predictor of IRHs. Statistically significant findings are emboldened.

### 3.4 Rates of IRH and ATEs according to treatment intent (ITT)

Given that both the NC+RC and the NCnoRC groups were intended for neoadjuvant treatment, we sought to compare the rates of IRHs and ATEs among these two combined groups as compared to those whom received AC. No differences in IRHs (3.7% vs 2.9% *p* = 0.44) nor ATEs (9.9% vs 9.7%, *p* = 1) were seen on univariate and multivariate analyses. Once again, on multivariate analyses, advancing age was strongly associated with ATE only but not IRHs. In particular, patients over age 75 demonstrated an adjusted risk of 1.85 compared to those less than 65 years (Table 4).

**TABLE 4:** Multivariate analysis of both ATEs and IRH within the ITT cohorts

Characteristics	ATE		IRH	
	HR (95%CI)	*p* value	HR (95%CI)	*p* value
ITT arm	1.06 (0.68‐1.62)	0.78	0.73 (0.30‐1.65)	0.45
Male Gender	1.45 (0.93‐2.37)	0.12	1.51 (0.68‐3.41)	0.32
Age 65‐74	**1.66 (1.05‐2.54)**	**0.02**	1.76 (0.83‐3.72)	0.14
Age >75	**1.85 (1.09‐2.79)**	**0.01**	1.86 (0.83‐4.16)	0.13
CI	1.08 (0.93‐1.27)	0.32	1.07 (0.81‐1.39)	0.62
Era 2 (2008‐)	1.06 (0.69‐1.64)	0.10	1.45 (0.75‐2.81)	0.27

Multivariate analyses for ITT cohorts. Advancing is associated with greater risk of ATE but not IRH. Statistically significant findings are emboldened.

## 4 DISCUSSION

Utilization of NC in MIBC is increasingly recognized as standard of care. In this analysis of the entire Province of Ontario over a 16‐year period, 29.6% of patients whom were planned to undergo cystectomy were also initiated on NC. This proportion is consistent with prior studies published using a similar dataset.^21^ In the United States, use of NC in conjunction with RC has varied widely, ranging from 12 to 57% from 2010 to 2015. It is still considered an underutilized therapy.^22^, ^28^ ^.^Possible explanations for a moderate level of completion could be a continued skepticism toward the risk/benefits of NC. Multidisciplinary tumor boards are shown to improve use of NC in other malignancies and may serve as an impetus for wider academic adoption of concerted meetings for bladder cancer patients.^29^, ^30^ Additionally, many patients are simply not eligible for this multimodal treatment due to competing comorbidities. Over this period, use of NC significantly increased from 3% to 35% suggesting a wider adoption and acceptance into current clinical practice (*p* < 0.001).

These data, for the first time, demonstrate that over one half of all patients who were given chemotherapy with the intent toward surgery, never actually received the potentially curative procedure. This proportion did decrease significantly (*p* < 0.001) over time to 20.5% by 2015, but remains a large proportion of patients. The sizable amount of patients whom commence chemotherapy and never make it to RC is an interesting group to hypothesize upon. These findings call into question the generalizability of prior publications in this space that have utilized the actual RC as a start point for cohort assembly.^19^, ^20^, ^21^, ^29^ When comparing predictors of initiation versus completion of NC, we see a significant difference in baseline characteristics. The NCnoRC cohort is older, harbors significantly greater life‐threatening comorbidities, and has a higher CI than those completing NC+RC (Table 1). We had initially hypothesized that perhaps these patients never make it to RC due to significant ATEs and IRH but in fact this was not the case on multivariate analysis. It is also plausible that patients with bulky nodal disease were included in the cohort. However, we are confident that our cohort assembly strategy has eliminated a vast majority of these patients as stipulated by no more than four cycles of chemotherapy as well as specifically eliminating patients coded as (cN2‐3) which represent bulky tumors. We thus hypothesize that NCnoRC either progress on treatment move toward palliative care or experience some other form of clinical deterioration that hinders the possibility of a safe or clinically warranted RC. Another possibility is that the chemotherapy intent coding is not accurate. We discount this possibility based upon prior data in breast cancer demonstrating that the Ontario chemotherapy data are accurate.^31^ As delays in completion of chemotherapy or surgery are associated with poor oncologic outcomes,^27^ further investigation is encouraged to best determine how to manage these challenging patients. In light of the fact that the RC‐only groups and NCnoRC groups were similarly balanced, perhaps upfront surgery should be considered as a best oncologic approach with an accepted increased risk of ATE. Clearly, more work is needed to better manage these elderly and comorbid patients (age ≥75, Charlson score ≥0.70).

Another interesting observation in this study revolves around rates of IRHs and ATEs. Contrary to our pre‐study biases, there was no correlation between timing of perioperative chemotherapy and rates of IRHs or ATEs. Interestingly, those receiving RC‐only had the highest rates of events (12.5%). Patients receiving RC‐only are significantly older, more comorbid, and have higher CI scores than those receiving multimodal treatment and thus explains these findings (Table 1). The impact of advancing age seems to be the most consistent risk factor for ATEs and IRHs across our dataset. Overall, these data suggest that pre‐existing age and comorbidity as the strongest driver of complications in managing patients requiring RC+/− chemotherapy. Of note, it is possible that a small subset of our patients in the RC‐only cohort was treated for BCG refractory or high risk non‐MIBC. However, population‐based studies do suggest that only 4.7–7.6% of all RC’s represent patients with this clinical scenario and would therefore not objectively alter these estimates.^32^, ^33^, ^34^, ^35^

A stubbornly high absolute IRH rate of 5% and ATE rate of 11.5% exists for all groups. As a compliment to this dilemma, prior studies have also demonstrated a 5.7% reoperation rate in this study population.^36^ Overall perioperative complications can reach as high as 58% within 90 days of RC^37^ and readmission rates as high as 25–40%^38^, ^39^ Clinical trials aimed at minimizing complications, such as routine anticoagulation or heart rhythm monitoring (TRACING trial),^40^ are encouraged in order to potentially avoid or detect ATEs at an earlier stage.

As in any study utilizing administrative datasets, certain limitations exist. As the data are not randomized, imbalances among treatment arms clearly exist and are the reason why we have not displayed any survival data (data not shown). Furthermore, detailed pathologic staging is not available. Although we acknowledge this limitation, our cohort is limited to patients with clinical N0/M0 disease. Unlike survival outcomes, substratification of stage within this narrow cohort is unlikely to be associated with differing risks for IRHs and ATEs. Secondly, we are aware that we did not stratify patients whom may have already been on anticoagulants/antiplatelet therapy. The reason why is that many patients in our cohort are less than 65 years of age and are not covered by the Ontario Drug benefit program (and thus data access on this population is not available in our Provinces databases).

This is the first study to our knowledge which compares the use and/or timing of perioperative chemotherapy in the non‐metastatic MIBC patient, to the risks of developing IRHs. In addition, we assess the rates of ATEs in this population according to the use/and or timing of perioperative chemotherapy. This study confirms that chemotherapeutic timing does not impact IRH nor ATE outcomes, as compared to advancing age, CI score, and modern Era selection. While our data demonstrate increased adoption and completion of NC+RC, a noteworthy 20% of patients in whom chemotherapy is initiated, fail to progress to the extirpative procedure. They do not possess higher age or CI than completers of NC+RC and as such, reasons for the NCnoRC proportion remains likely related to oncologic progression, other deleterious events, or patient preference.

These data report on the utilization of perioperative chemotherapy over the past generation. The introduction of multidisciplinary bladder clinics and in the near future the adjunct of molecular biomarkers presents an opportunity to better select patients whom are likely to benefit from perioperative systemic treatment. Novel therapies with demonstrated efficacy in advanced urothelial carcinoma such as immunotherapeutics and nectin‐4 targeting may, in the future represent a safer and more effective perioperative strategy.^41^, ^42^, ^43^

## 5 CONCLUSION

In the Province of Ontario, trends suggest an increase in the adoption of NC from 3% in 2002 to 35% in 2015 (*p* < 0.01). RC‐only still remains the largest utilized treatment approach at 54% and a substantial proportion of patients, particularly the elderly and comorbid, fail to progress to surgical intervention (NCnoRC), although this number has substantially improved (50.3% to 20.5%). Rates of ATEs and IRHs remain stubbornly high, are not associated with timing of chemotherapy, but are rather associated to unmodifiable patient risk factors; notably older age (*p* < 0.001) and in certain instances higher CI score. Research is encouraged to better identify strategies to limit the rates of IRH and ATE in this unique population.

## CONFLICT OF INTEREST

There are no conflicts of interest.

## ETHICAL STATEMENT

Ethical approval was sought from an institutional review board (REB at UHN) and approved.

AUTHOR CONTRIBUTIONS

Tarik Benidir conceived and designed the analysis, collected the data, contributed data or analysis tools, performed the analysis, and wrote the paper. Jaime Herrera‐Caceres contributed data or analysis tools and performed the analysis. Christopher C.J Wallis conceived and designed the analysis. Katherine Lajkosz contributed data or analysis tools. Neil Fleshner conceived and designed the analysis, contributed data or analysis tools, performed the analysis, and wrote the paper.

## Supporting information

Appendix S1

## Data Availability

Raw data were generated at the Institute for Clinical and Evaluative Sciences. Derived data supporting the findings of this study are available from the corresponding author upon request and should also go through the regulatory/patient safety approval board at this aforementioned institution.

## References

[1] Sherif A , Rintala E , Mestad O , et al. Neoadjuvant cisplatin‐methotrexate chemotherapy for invasive bladder cancer—nordic cystectomy trial 2. Scand J Urol Nephrol. 2002;36:419‐425. 12623505 10.1080/003655902762467567

[2] Sherif A , Holmberg L , Rintala E , et al. Neoadjuvant cisplatinum based combination chemotherapy in patients with invasive bladder cancer: a combined analysis of two Nordic studies. Eur Urol. 2004;45:297‐303. 15036674 10.1016/j.eururo.2003.09.019

[3] Griffiths G , Hall R , Sylvester R , et al. International phase III trial assessing neoadjuvant cisplatin, methotrexate, and vinblastine chemotherapy for muscle‐invasive bladder cancer: long‐term results of the BA06 30894 trial. J Clin Oncol. 2011;29:2171‐2177. 21502557 10.1200/JCO.2010.32.3139 PMC3107740

[4] Grossman HB , Natale RB , Tangen CM , et al. Neoadjuvant chemotherapy plus cystectomy compared with cystectomy alone for locally advanced bladder cancer. N Engl J Med. 2003;349:859‐866. 12944571 10.1056/NEJMoa022148

[5] Advanced Bladder Cancer (ABC) Meta‐analysis Collaboration. Neoadjuvant chemotherapy in invasive bladder cancer: update of a systematic review and meta‐analysis of individual patient data. Eur Urol 2005;48(2):202‐206;discussion 205–6 15939524 10.1016/j.eururo.2005.04.006

[6] Skinner DG , Daniels JR , Russell CA , et al. The role of adjuvant chemotherapy following cystectomy for invasive bladder cancer: a prospective comparative trial. J Urol 1991;145(3):459‐464; discussion 464–7. 1997689 10.1016/s0022-5347(17)38368-4

[7] Stockle M , Meyenburg W , Wellek S , et al. Adjuvant polychemotherapy of nonorgan‐confined bladder cancer after radical cystectomy revisited: long‐term results of a controlled prospective study and further clinical experience. J Urol. 1995;153:47‐52. 7966789 10.1097/00005392-199501000-00019

[8] Lehmann J , Franzaring L , Thuroff J , et al. Complete long‐term survival data from a trial of adjuvant chemotherapy vs control after radical cystectomy for locally advanced bladder cancer. BJU Int. 2006;97:42‐47. 16336326 10.1111/j.1464-410X.2006.05859.x

[9] Cognetti F , Ruggeri EM , Felici A , et al. Adjuvant chemotherapy with cisplatin and gemcitabine versus chemotherapy at relapse in patients with muscle‐invasive bladder cancer submitted to radical cystectomy: an Italian, multicenter, randomized phase III trial. Ann Oncol. 2012;23:695‐700. 21859900 10.1093/annonc/mdr354

[10] Stadler WM , Lerner SP , Groshen S , et al. Phase III study of molecularly targeted adjuvant therapy in locally advanced urothelial cancer of the bladder based on p53 status. J Clin Oncol. 2011;29:3443‐3449. 21810677 10.1200/JCO.2010.34.4028 PMC3164246

[11] Bagrodia A , Sukhu R , Winer AG . Incidence and effect of thromboembolic events in radical cystectomy patients undergoing preoperative chemotherapy for muscle‐invasive bladder cancer. Clin Genitourin Cancer. 2017;16(1):E113–E120. pii: S1558–7673(17)30232‐X. 10.1016/j.clgc.2017.07.022 PMC6053335 28866245

[12] Boeri L , Soligo M , Frank I , et al. Delaying radical cystectomy after neoadjuvant chemotherapy for muscle‐invasive bladder cancer is associated with adverse survival outcomes. Eur Urol Oncol. 2019;2:390‐396. 31277775 10.1016/j.euo.2018.09.004

[13] Nendaz M , Spirk D , Kucher N , et al. Multicentre validation of the Geneva Risk Score for hospitalised medical patients at risk of venous thromboembolism. Ex‐plicit ASsessment of Thromboembolic RIsk and Prophylaxis for Medical PA‐ Tients in SwitzErland (ESTIMATE). Thromb Haemost. 2014;111(3):531–538. 24226257 10.1160/TH13-05-0427

[14] Zareba P , Duivenvoorden WCM , Pinthus JH . Thromboembolism in patients with bladder cancer: incidence, risk factors and prevention. Bladder Cancer. 2018;4(2):139‐147. 29732385 10.3233/BLC-170146 PMC5929309

[15] Navi BB , Reiner AS , Kamel H , et al. Risk of arterial thromboembolism in patients with cancer. J Am Coll Cardiol. 2017;70(8):926‐938. 28818202 10.1016/j.jacc.2017.06.047 PMC5667567

[16] Fantony JJ , Gopalakrishna A , Noord MV , et al. Reporting bias leading to discordant venous thromboembolism rates in the United States versus non‐US countries following radical cystectomy: a systematic review and meta‐analysis. Eur Urol Focus. 2016;2(2):189‐196. 10.1016/j.euf.2015.09.003 PMC4941825 28723534

[17] Seng S , Liu Z , Chiu SK , et al. Risk of venous thromboembolism in patients with cancer treated with cisplatin: a systematic review and meta‐analysis. J Clin Oncol. 2012;30(35):4416‐4426. 23150697 10.1200/JCO.2012.42.4358

[18] Proverbs‐Singh T , Chiu SK , Liu Z , et al. Arterial thromboembolism in cancer patients treated with cisplatin: a systematic review and meta‐analysis. J Natl Cancer Inst. 2012;104(23):1837‐1840. 23093559 10.1093/jnci/djs435

[19] Brennan K , Karim S , Doiron RC , et al. Venous thromboembolism and peri‐operative chemotherapy for muscle‐invasive bladder cancer: a population‐based study. Bladder Cancer. 2018;4:419‐428. 30417053 10.3233/BLC-180184 PMC6218104

[20] Duivenvoorden WC , Daneshmand S , Canter D , et al. Incidence, characteristics and implications of thromboembolic events in patients with muscle invasive urothelial carcinoma of the bladder undergoing neoadjuvant chemotherapy. J Urol. 2016;196:1627‐1633. 27312316 10.1016/j.juro.2016.06.017

[21] Zareba P , Patterson L , Pandya R , et al. Thromboembolic events in patients with urothelial carcinoma undergoing neoadjuvant chemotherapy and radical cystectomy. Urol Oncol. 2014;32:975‐980. 25027682 10.1016/j.urolonc.2014.03.025

[22] Cowan NG , Chen Y , Downs TM , et al. Neoadjuvant chemotherapy use in bladder cancer: a survey of current practice and opinions. Adv Urol. 2014;2014:746298. 24982672 10.1155/2014/746298 PMC4058463

[23] Khorana AA , Connolly GC . Assessing risk of venous thromboembolism in the patient with cancer. J Clin Oncol. 2009;27:4839‐4847. 19720906 10.1200/JCO.2009.22.3271 PMC2764392

[24] Doiron RC , Booth CM , Wei X , et al. Risk factors and timing of venous thromboembolism after radical cystectomy in routine clinical practice: a population‐based study. BJU Int. 2016;118(5):714‐722. 10.1111/bju.13443 26950039

[25] Chu AT , Holt SK , Wright JL . Delays in radical cystectomy for muscle‐invasive bladder cancer. Cancer;125(12):2011‐2017. 10.1002/cncr.32048 30840335

[26] Russell B , Liedberg F , Khan MS . A systematic review and meta‐analysis of delay in radical cystectomy and the effect on survival in bladder cancer patients. Eur Urol Oncol. 2020;3:239‐249. 31668714 10.1016/j.euo.2019.09.008

[27] Boeri L , Soligo M , Karnes J , et al. Delaying radical cystectomy after neoadjuvant chemotherapy for muscle‐invasive bladder cancer is associated with adverse survival outcomes. Eur Urol Oncol. 2019;2(4):390‐396. 31277775 10.1016/j.euo.2018.09.004

[28] Clinton TN , Wiseman M , Walasek A . Commentary: underutilization of curative‐intent therapy for patients with muscle‐invasive bladder cancer in Sweden mimics the United States. Transl Androl Urol. 2019;8(Suppl 5):S542‐S545. 32042642 10.21037/tau.2019.12.22 PMC6989840

[29] Gotto G , Melissa A , Budgell S , et al. Predictors of referral for neoadjuvant chemotherapy prior to radical cystectomy for muscle‐invasive bladder cancer and changes in practice over time. Can Urol Assoc J. 2015;9(7–8):236‐241. 26316905 10.5489/cuaj.2722 PMC4537332

[30] Bumm R , Feith M , Lordick F , et al. Impact of multidisciplinary tumor boards on diagnosis and treatment of esophageal cancer. Euro Surg. 2007;39:136‐140.

[31] Weerasinghe A , Smith CR , Majpruz V , et al. Validity of administrative databases in comparison to medical charts for breast cancer treatment data. J Cancer Epidemiol. 2018;2018:9218595. 29861727 10.1155/2018/9218595 PMC5976924

[32] Warren M. , Kolinsky M. , Canil C.M. Canadian Urological Association/Genitourinary Medical Oncologists of Canada consensus statement: management of unresectable locally advanced and metastatic urothelial carcinoma. Can Urol Assoc J 2019;13(10):318–327. Epub ahead of print. 10.5489/cuaj.6015 PMC6788915 31059420

[33] Kikuchi E. , Hayakawa N. , Fukumoto K. Bacillus Calmette–Guérin‐unresponsive non‐muscle‐invasive bladder cancer: Its definition and future therapeutic strategies. Int J Urol 2020;27(2):108‐116. 31793703 10.1111/iju.14153

[34] Tilki D. , Reich O. , Svatek R.S. Characteristics and outcomes of patients with clinical carcinoma in situ only treated with radical cystectomy: an international study of 243 patients. J Urol. 2010;183(5):1757–1763. 20299059 10.1016/j.juro.2010.01.025

[35] Huguet J , Gaya J , Sabaté S Radical cystectomy in patients with non‐muscle invasive bladder cancer who fail BCG therapy. Actas Urol Esp 2010;34(1):63‐70. 20223134

[36] Lyon TD , Boorjian SA , Shah PH , et al. Comprehensive characterization of perioperative reoperation following radical cystectomy. Urol Oncol. 2019;37(4):292.e11‐292. 10.1016/j.urolonc.2018.11.023 30679081

[37] Hautmann RE , de Petriconi RC , Volkmer BG . Lessons learned from 1,000 neobladders: the 90‐day complication rate. J Urol. 2010;184:990‐994. 20643429 10.1016/j.juro.2010.05.037

[38] Al‐Daghmin A , Aboumohamed A , Din R , et al. Readmission after robot‐assisted radical cystectomy: outcomes and predictors at 90‐day follow‐up. Urology. 2014;83(2):350‐356. 24468509 10.1016/j.urology.2013.09.056 PMC4431627

[39] McIntosh AG , Li T , Ito T , et al. WBC associates with readmission following cystectomy. Bladder Cancer. 2017;3:95‐103. 28516154 10.3233/BLC-160088 PMC5409152

[40] Downey C , Ng S , Jayne D , et al. Reliability of a wearable wireless patch for continuous remote monitoring of vital signs in patients recovering from major surgery: a clinical validation study from the TRaCINg trial. BMJ Open. 2019;9(8):e031150. 10.1136/bmjopen-2019-031150 PMC6701670 31420399

[41] Balar AV , Galsky MD , Rosenberg JE . Atezolizumab as first‐line therapy in cisplatin‐ineligible patients with locally advanced and metastatic urothelial carcinoma: a single‐arm, multicentre, phase 2 trial. Lancet. 2017;389(10064):67‐76. 27939400 10.1016/S0140-6736(16)32455-2 PMC5568632

[42] Bellmunt J , de Wit R , Vaughn DJ , et al. Pembrolizumab as Second‐Line Therapy for Advanced Urothelial Carcinoma. N Engl J Med 2017;376(11):1015‐1026. 28212060 10.1056/NEJMoa1613683 PMC5635424

[43] Rosenberg JE , O’Donnell PH , Balar AV , et al. Pivotal trial of enfortumab vedotin in urothelial carcinoma after platinum and anti‐programmed death 1/programmed death ligand 1 therapy. J Clin Onc 2019;37(29):2592‐2600. 10.1200/JCO.19.01140 PMC6784850 31356140

