# Supplementary material for: Population‐based analysis of perioperative chemotherapy use, interventions requiring hospitalization and atheroembolic events among patients with non‐metastatic muscle‐invasive bladder cancer
Source: Cancer Med. 2021 Mar 12;10(8):2636–44. doi: 10.1002/cam4.3805 (PMC8026920; doi:10.1002/cam4.3805)
Supplement: Supplementary file 1 — Appendix S1 [file CAM4-10-2636-s001.docx]

1. **Lists of Arterial and venous thromboembolic events with their database codes**
   - ICD-10:
     - I74.0-I74.9 Arterial Embolism and thrombosis
     - I75.011-I75.019– Atheroembolism of upper extremity
     - I75.021-I75.029 – Atheroembolism of lower extremity
     - I75.81-I75.89 – Atheroembolism of other sites
     - I63.00-I63.9 – Cerebral infarction
     - I61.0-I61.9 – non traumatic intracerebral hemorrhage
     - I21.0-I21.9 – Myocardial infarction
     - I22.0-I22.9- STEMI and nSTEMI
     - K55.01-K55.069 – Mesenteric embolism and thrombosis
     - I26.01-I26.9 – pulmonary embolism
     - N28 - Renal embolism and thrombosis –
     - H34.01-H34.9 - Retinal embolism and thrombosis
     - I82.210 – Acute embolism and thrombosis of superior vena cava
     - I82.220 – Acute embolism and thrombosis of inferior vena cava
     - I82.290 – Acute ambolism and thrombosis of other thoracic veins
     - I82.3 – Acute embolism and thrombosis of renal vein
     - I82.4- Acute embolism and thrombosis of deep veins of lower extremity
     - (I82.401, I82.402, I82.403, I82.409, I82.411, I82.412, I82.413, I82.419, I82.421, I82.422, I82.423, I82.429, I82.431, I82.432, I82.433, I82.439, I82.441, I82.442, I82.443, I82.449, I82.491, I82.492, I82.493, I82.499, I82.4Y1, I82.4Y2, I82.4Y3, I82.4Y9, I82.4Z1, I82.4Z3, I82.4Z9)–
     - I82.601, 602, 603, 609- Acute embolism and thrombosis of veins of upper extremity
     - I82.A11, A12, A13, A19 – Acute embolism and thrombosis of axillary veins
     - I82.B11, B12, B13, B19 – Acute embolism and thrombosis of subclavian vein
     - I82.C11, C12, C13, C19- Acute embolism and thrombosis of internal jugular vein
     - I82.90 – Embolism and thrombosis of unspecified veins.
2. **Lists of Interventions Requiring Hospitalization with their database codes**

- ATE/VTE requiring procedural interventions
  - **ICD-10**
    - Coronary angioplasty (Z98.61)
  - **Cardiac/Vascular (OHIP)**
    - G297 (Angiography)
    - G509 (bypass graft angiogram)
    - Z442 (selective coronary catheterization) – both arteries
    - G263 (selective coronary catheterization) – with other drug interventional studies
    - Z434 (Transluminal coronary angioplasty) – one or more sites on a single major vessel
    - G262 (Transluminal coronary angioplasty) – Each additional major vessel
    - G298 – Coronary angioplasty stent
    - Z444, Z435– Insertion of permanent endocardial electrode
    - Z429, R751, R752 – Other electrode interventions
    - R701-R704 - Ventricular assisted devices
    - R753, R761 – Insertion of a defibrillator
    - R741 – Coronary artery endarterectomy
    - R742-R743 – Coronary artery repair
    - E667, E627 – Ruptured thoracic or abdominal aortic aneurysm
    - R814,R813, R867 – Embolectomy or thrombectomy
    - R809 – Fem-pop endarterectomy
    - R878-879 – Recanalization of fem/pop/tibial arterial occlusive disease
